# Assessment of nonnegative matrix factorization algorithms for electroencephalography spectral analysis

**DOI:** 10.1186/s12938-020-00796-x

**Published:** 2020-07-31

**Authors:** Guoqiang Hu, Tianyi Zhou, Siwen Luo, Reza Mahini, Jing Xu, Yi Chang, Fengyu Cong

**Affiliations:** 1 School of Biomedical Engineering, Faculty of Electronic Information and Electrical Engineering, Dalian University of Technology, Dalian, China; 2 Center for Cognition and Neuroergonomics, State Key Laboratory of Cognitive Neuroscience and Learning, Beijing Normal University at Zhuhai, Zhuhai, China; 3 Affiliated Zhongshan Hospital of Dalian University, Dalian, China; 4 Department of Neurology and Psychiatry, First Affiliated Hospital, Dalian Medical University, Dalian, China; 5 School of Artificial Intelligence, Faculty of Electronic Information and Electrical Engineering, Dalian University of Technology, Dalian, China; 6 Key Laboratory of Integrated Circuit and Biomedical Electronic System, Dalian University of Technology, Dalian, Liaoning China; 7 Faculty of Information Technology, University of Jyvaskyla, Jyvaskyla, Finland

**Keywords:** Nonnegative matrix factorization, Stability, Clustering, EEG

## Abstract

**Background:**

Nonnegative matrix factorization (NMF) has been successfully used for electroencephalography (EEG) spectral analysis. Since NMF was proposed in the 1990s, many adaptive algorithms have been developed. However, the performance of their use in EEG data analysis has not been fully compared. Here, we provide a comparison of four NMF algorithms in terms of accuracy of estimation, stability (repeatability of the results) and time complexity of algorithms with simulated data. In the practical application of NMF algorithms, stability plays an important role, which was an emphasis in the comparison. A Hierarchical clustering algorithm was implemented to evaluate the stability of NMF algorithms.

**Results:**

In simulation-based comprehensive analysis of fit, stability, accuracy of estimation and time complexity, hierarchical alternating least squares (HALS) low-rank NMF algorithm (lraNMF_HALS) outperformed the other three NMF algorithms. In the application of lraNMF_HALS for real resting-state EEG data analysis, stable and interpretable features were extracted.

**Conclusion:**

Based on the results of assessment, our recommendation is to use lraNMF_HALS, providing the most accurate and robust estimation.

## Background

Nonnegative matrix factorization (NMF) is a low-rank approximation method where both the data and the estimated low-rank factors are constrained to be nonnegative [1]. The method has been widely applied for data analysis and signal processing [2–4]. NMF was first introduced as positive matrix factorization (PMF) by Paatero and Tapper [5] and it was popularized from the application of face recognition by Lee and Seung [6]. Since it was proposed in the 1990s, many adaptive algorithms have been developed [2, 7]. Among numerous algorithms of NMF, multiplicative updating rule-based algorithm [8] is the most popular one. Besides, more efficient algorithms based on hierarchical alternating least squares (HALS) method and low-rank approximation were also proposed. However, the performance of NMF in the implementation of EEG analysis has not been fully compared.

Electroencephalography (EEG) spectral studies deepen the understanding of the neurophysiological processes. It has been shown that the theta frequency band (3–8 Hz) reflects the general motor activity and the delta activity (0.5–3 Hz) has relationship with error-related processing [9, 10]. In working memory tasks, the global field theta synchronization significantly correlated with the blood oxygen level dependent (BOLD) signal [11]. It is also shown that gamma power (60–80 Hz) of EEG positively correlated with BOLD fluctuation and alpha (8–13 Hz) and beta (14–30 Hz) power negatively correlated with BOLD fluctuation [12].

Insomnia is a clinically common and frequent disease. The patient complains of poor sleep and accompanied by impaired day time functions, such as fatigue, lack of energy, cognitive decline, and emotional disorders [13, 14]. Currently, researchers found that the factors that induce insomnia mainly include age, personality, anxious personality, family and personal history of insomnia, genetic factors, etc. [15–17]. The main pathophysiological mechanism of patients with primary insomnia is excessive awakening [18–20]. In order to confirm the theory of excessive awakening, several studies were conducted on autonomic nerve activity, neuroendocrine, neuroimmunology, neuroelectrophysiology, and neuroimaging in patients with insomnia [21–23]. The spectral EEG was used to analyze the sleep electroencephalogram of patients with primary insomnia [24, 25], and it was found that the high-frequency waves of patients with insomnia were more active than normal people, including the period pre-sleep [26, 27], the wake–sleep transition period [28], and the sleep period [27, 29, 30]. Studies have shown that high-frequency brain waves are related to sensory processing, memory formation, and consciousness perception, and the active response of high-frequency brain waves increases cortical arousal levels [31]. Therefore, this study conducted a spectral analysis of the EEG of insomnia patients at rest to further explore the pathophysiological mechanism of primary insomnia.

NMF has been successfully used in the analysis of EEG spectral analysis. The components resulting from the NMF retain the spectral features hence they are interpretable from a physical or physiological point of view. For example, EEG large-scale network was constructed with NMF [32]. NMF was also used to estimate time and frequency components to predict epileptic seizures [33]. Both for static and dynamic functional connectivity, interpretable features also could be extracted with NMF [34, 35]. In the field of brain computer interface (BCI), NMF can obtain components with distinguishing features [36–38]. Several studies used features extracted with NMF to improve the discrimination of different participants [39–42]. Gruve et al. used NMF to extract the weight of the EEG channels to improve the accuracy of motor imagery detection [43] and classification of eye states [44]. With the successful application of NMF algorithm on EEG data, more and more NMF algorithms are constantly emerging [45–48]. In these studies, researchers used different NMF algorithms to analyze EEG data. However, the performance of NMF algorithms has not been fully compared.

Compared with independent component analysis (ICA) [49] and principal component analysis (PCA), NMF identified more meaningful and explainable components in practical application. Compared with PCA, the results of NMF have explicable physical meaning and they are consistent with the brain intuitive perception [38]. Compared with ICA, some interesting components with higher distinguishing ratio were extracted by NMF [50].

The importance of stability of algorithms is self-evident for scalp EEG data analysis. Stability is a common problem of adaptive algorithms. Same to other blind sources separation (BSS) methods such as ICA, NMF also faces the same issue. Theoretically, just like the stability analysis of ICA [51, 52], the uniqueness of NMF could be verified by geometric interpretation [2, 49]. However, in the practical applications, algorithms may converge to local optima, which has been theoretically analysis with Lyapunov’s first and second methods [53]. Thus, the stability of algorithms would be the most important aspect in the assessment of NMF algorithms.

In this study, the performance of four NMF algorithms was compared in terms of accuracy of estimation, stability and time complexity of algorithms with simulated data. The algorithm with excellent performance in simulation data was applied to real resting-state EEG data analysis. Based on stability analysis method, stable and interpretable features were extracted.

## Simulation and results

**Fig. 1:**
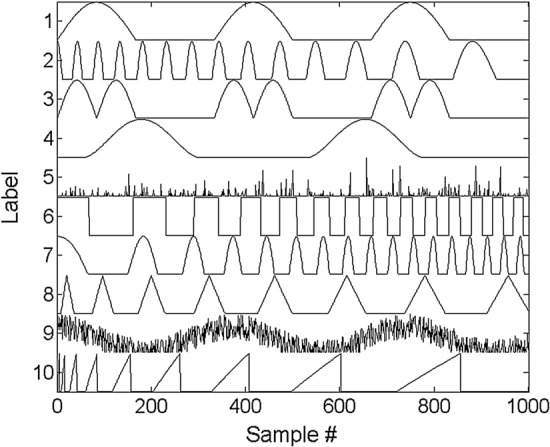
Waveforms of 10 time-series for H in the NMF model

Given a signal $H \in {\mathbb{R}}_{ + }^{10\; \times \;1000}$ as component matrices shown in Fig. 1, we generated a mix matrix $W \in {\mathbb{R}}_{ + }^{10 \times 64}$. Then we constructed $V^{*} = W^{T} H \in {\mathbb{R}}_{ + }^{64 \times 1000}$ and $V = V^{*} + E$, where $E$ denoted the independent noise and $V$ denoted 64-channel simulated time-series EEG data. Signal noise ratio (SNR) was used to quantitative describe the quality of data. In this simulation, compared the performance of four NMF algorithms (NMF_MU, HALS, lraNMF_MU, lraNMF_HALS) were compared. The number of extracted components $r$ was chosen as 10. The same algorithm was run 50 times. 500 components in total were clustered. All four algorithms were run in Matlab under the Window 10 system (Intel Xeon CPU 3.5 GHz and 32 GB of Random Access Memory (RAM)).

### Simulated data with SNR = 20 dB

**Fig. 2:**
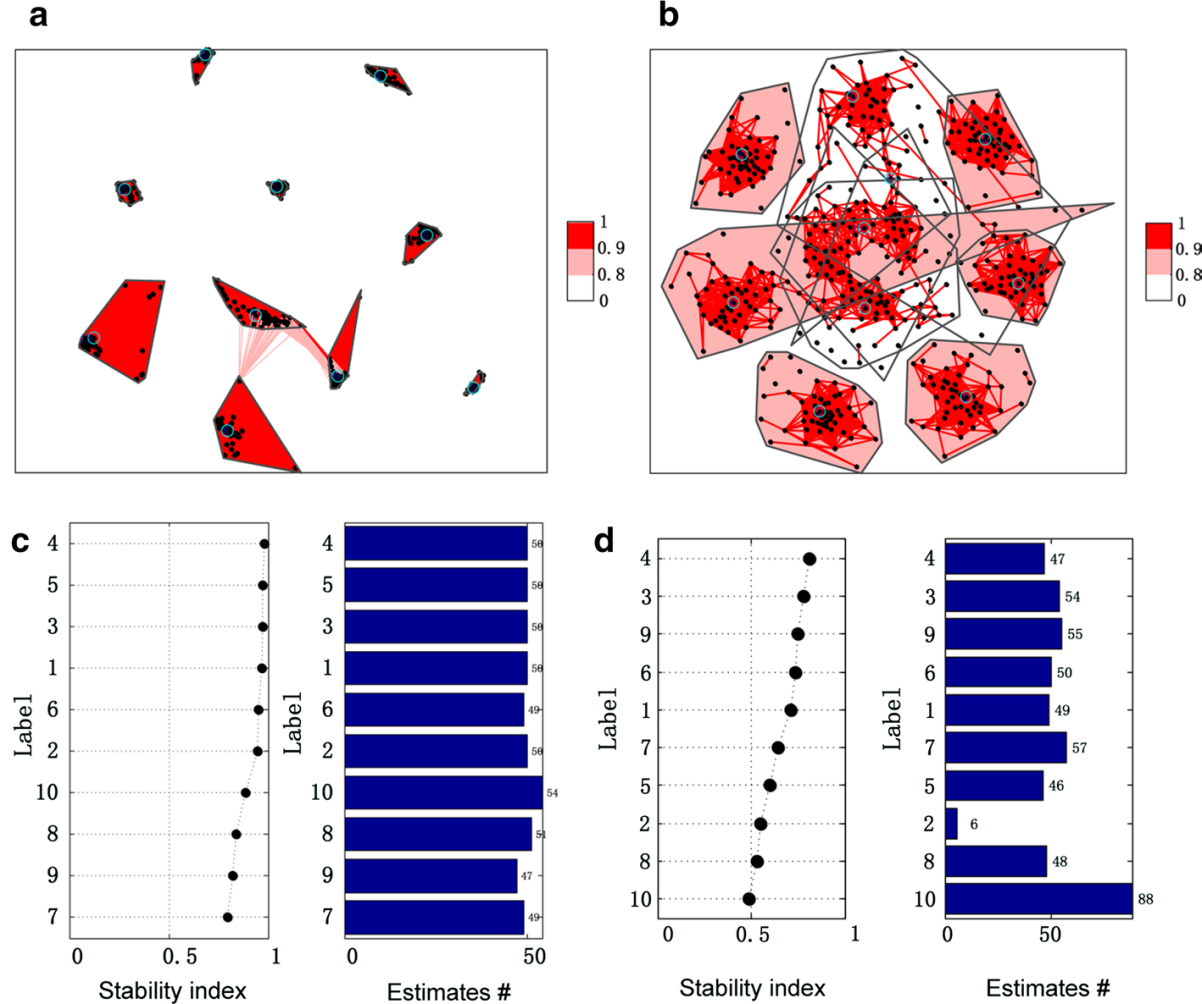
Clustering 500 extracted components from 50 runs of NMF with 10 components in each run: **a** inner similarity of each cluster for HALS; **b** inner similarity of each cluster for NMF_MU; **c** stability index ($I_{q}$) and the number of components in each cluster for HALS; **d** stability index ($I_{q}$) and the number of components in each cluster for NMF_MU

**Fig. 3:**
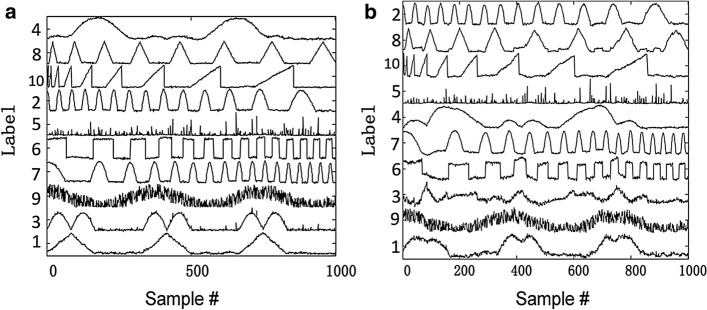
Illustration of 10 extracted components by two NMF algorithms. **a** Components extracted by HALS; **b** components extracted by NMF_MU

Simulated data with SNR of 20 dB was used for demonstrating the clustering related results. The 10 estimated sources and the clustering results of all 500 components estimated with HALS and NMF_MU algorithms are shown in Fig. 2. Ideally, the number of components in each cluster equals to the number of times that NMF was run (50 at here), under the condition that the number of clusters is same with the number of extracted components (10 at here). Obviously, HALS outperformed NMF_MU from the view of the stability of extracted components. In Fig. 2, the denser the cluster, the more stable of the components, extracted by an NMF algorithm, are. The purpose of the NMF algorithm is to accurately estimate the potential components in the data. Hence, it is necessary to check the accuracy of estimated sources. Here, readers are guided to have an intuitive feeling of the accuracy of estimation. The quantitative comparison in terms of estimation accuracy would be shown in the next section. The centroid of each cluster was selected as the component extracted by NMF as ICASSO [54] applied. Figure 3 shows the waveforms of 10 extracted components by two NMF algorithms. By visual inspection, HALS outperformed NMF_MU for estimating the sources.

Simulated data with multiple SNR

**Fig. 4:**
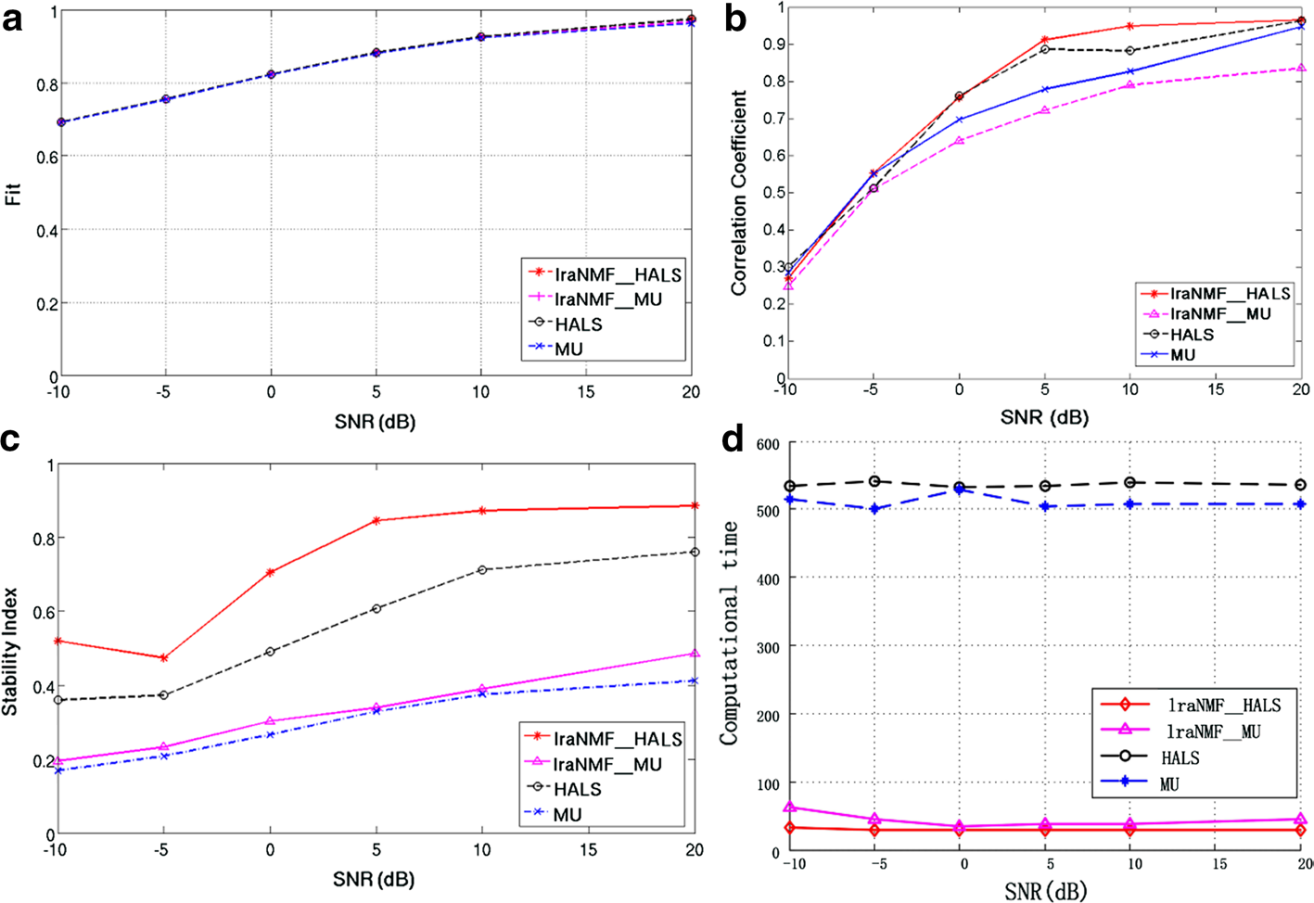
Results of four NMF algorithms with different SNR: **a** fit of NMF model; **b** correlation coefficient between the estimated source and the real source; **c** stability of an extracted component by NMF using clustering

In order to test the performance of different NMF algorithms under different SNR, Fig. 4 illustrates the results with SNR ranging from z− 10 dB to 20 dB with the step of 5 dB. Figure 4a shows that fits of four different NMF algorithms were very similar to one another. Figure 4b reveals the estimation accuracy of four algorithms. The estimation accuracy is measured with average correlation coefficient (Pearson correlation was used in this study) cross-ground truth and estimated component. The four algorithms yielded different correlation coefficients, which mean the accuracy of the estimation of four algorithms is different. The situation is a little bit different across SNR. When SNR is lower than − 5 dB, the estimation accuracy is mainly dominated with signal quality, the accuracy of all four algorithms is very low. As the SNR increases, the accuracy of the estimated components also increases. When SNR large than 0 dB, the performance of four algorithm begins to differ in terms of estimation accuracy. By comparison, it can be found that the estimated component of lraNMF_HALS is the most accurate, followed by HALS, followed by lraNMF_MU, and MU is the worst. Figure 4c presents the mean over 10 $I_{q}$ of 10 components extracted by algorithm for each SNR and the Iq indicates the stability of the components over multiple runs of NMF. The stability of the extracted components of four NMF algorithms was very different. Figure 4d illustrates the computation time of four algorithms for the same data. The difference in computational time mainly is decided by the difference of iteration between algorithms and time complexity of the algorithms, which would be introduced in “Methods” section. The result of computation time shows that lraNMF_HALS needs less time to converge. Evidently, bigger correlation coefficient indicates better estimation of sources in Fig. 4b and the higher $I_{q}$ implies better stability of extracted components in Fig. 4c. Based on comprehensive analysis of *fit*, stability, accuracy of estimation and time complexity, lraNMF_HALS outperformed the other three NMF algorithms. The algorithm would be used in the application of real EEG data analysis.

## Real EEG results

**Fig. 5:**
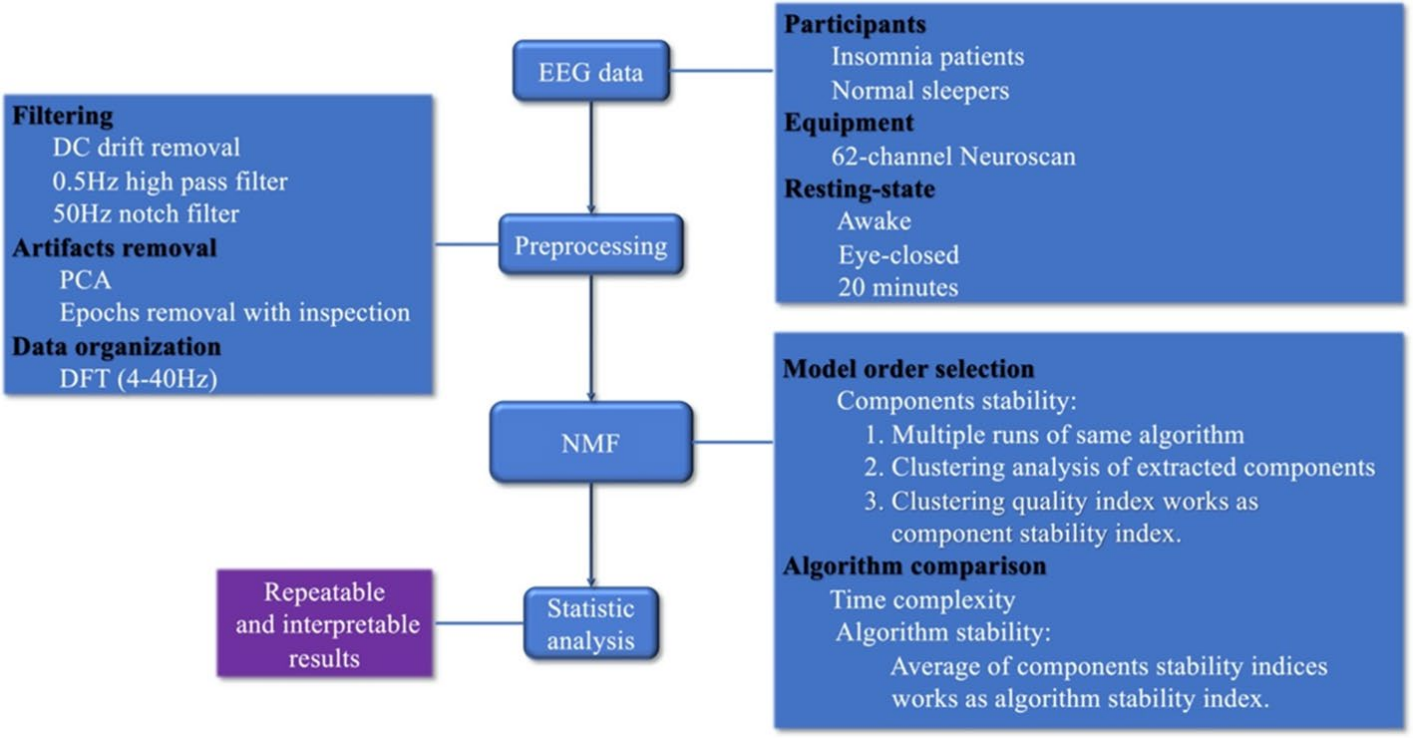
Flowchart of application of NMF algorithm to EEG data analysis

In this section, the application of NMF algorithm to EEG data analysis was introduced. The stability-validating method was extended used not only for algorithm stability evaluation, but also used for determination of number of extracted components. The flowchart of data processing is illustrated in Fig. 5. After EEG data were collected, standard EEG preprocessing procedure was applied. It includes data filtering and artificial removal. Then the EEG data were transformed into frequency domain, which would be fed into NMF algorithm. Performance of several NMF algorithms was then evaluated in terms of algorithm stability and time complexity. Next, the model order of NMF algorithm was selected in terms of reproducibility of estimated components. Based on the above rigorous steps, repeatable and interpretable components were finally obtained. The specific process of each step will be described in the following content.

### Data description

To evaluate the performance of four NMF algorithms, we tested it on EEG dataset which was recorded at The First Affiliated Hospital of Dalian Medical University. 16 primary insomnia patients and 17 normal sleepers were included in the study. Gender, age and education were matched between the two groups. All patients fit criteria for primary insomnia in DSM-IV. Patients and normal sleepers are required to complete the Pittsburgh Sleep Quality Index Scale (PSQI), Insomnia Severity Scale (ISI), Hyper-arousal Scale (HAS), Hamilton Anxiety Scale (HAMA) and Hamilton Depression Scale (HRSD-17) for clinical assessment. Continuous EEG recordings were digitally obtained using a 62-channel Neuroscan SynAmps2 brain electrical physiological instrument, EEG were recorded in a resting awake condition with the eyes closed for 20 min.

### Data preprocessing

The EEG data were sampled at 1000 Hz for recording. Subsequently, the data were resampled at 500 Hz. DC drifts were removed using 0.5 Hz high-pass filter and 50 Hz notch filters were applied to minimize line noise artifacts. PCA was used to determine linear components associated with eye blinks and saccades. Epochs with strong eye movement or other movement artifacts were manually removed by inspection.

For all further analyses, EEG signals were processed with the method of discrete Fourier transform (DFT) which transform the time series into frequency domain. We cut 4–40 Hz of our interest frequency band. Thus, the sizes of preprocessed data are 73 (frequency bins) by 62 (channels) by 33 (2 groups with 16 primary insomnia patients and 17 health control). Then we transform the three-dimensional matrix from the EEG data of each subject into the matrix (channels × frequency bins by samples, $62 \times 2409\left( {73 \;{\text{by}}\; 33} \right)$) and used it as input to the space-by-frequency decomposition. Based on these processing, the elements in the matrix that would be fed into NMF are nonnegative.

### Determination of the model order

Here, lraNMF_HALS was run 50 times with random initialization with the model order (number of extracted components) ranging from 2 to 30. Coefficient matrix was used to evaluate the stability of algorithms.

**Fig. 6:**
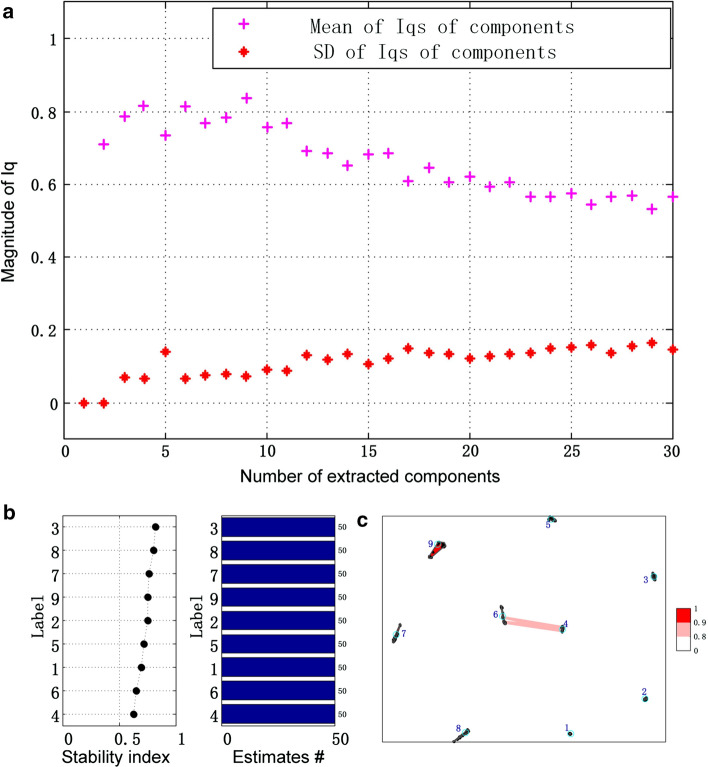
**a** Stability of lraNMF_HALS decomposition for each component (the pink mark denoted by the averaged $I_{q}$ and the red mark denoted by standard deviation of $I_{q}$); **b** clustering 450 extracted components from 50 runs of NMF with 9 components in each run, stability index (${\text{I}}_{\text{q}}$) and the number of components in each cluster for lraNMF_HALS; **c** inner similarity of each cluster for lraNMF_HALS

Figure 6a shows the stability (denoted by $I_{q}$) of lraNMF_HALS decomposition for each component. Indeed, each $I_{q}$ in Fig. 6a is the averaged $I_{q}$ for different components. It could be found that when the number of extracted components equals 9, the algorithm will be the most stable. As mentioned earlier, except the stability index of NMF decomposition, it is also necessary to check Fig. 6b, c and ensure whether the clustering result is good enough or not.

Different algorithms took different time finishing 50 times runs. NMF_MU took 1157.007s. HALS took 1173.178s. lraNMF_MU took 106.010s. lraNMF_HALS took 72.315s. Obviously, lraNMF_HALS took shortest time to complete the mission of decomposition. The stability indexes of four algorithms are also different. The stability index of NMF_MU is 0.760. The stability index of HALS is 0.717. The stability index of lraNMF_MU is 0.622. The stability index of lraNMF_HALS is 0.833. Obviously, as the result of simulation data, lraNMF_HALS is the most stable algorithm for the data. So, in the following subsection only the results of lraNMF_HALS would be introduced.

### Feature selection

After a matrix is decomposed by lraNMF_HALS with 9 components, the features need to be further filtrated. Usually, the criteria of feature selection are determined by the purpose of further feature analysis. As we know that the collected EEG data consist of brain activities of no interest, brain activities of interest, and interference as well as noise. Therefore, even though artifacts are removed in the data preprocessing phase, the features of interest and features of no interest may also be extracted by NMF. Therefore, feature selection step is necessary to choose the features of brain activities of interest for further analysis [55].

**Fig. 7:**
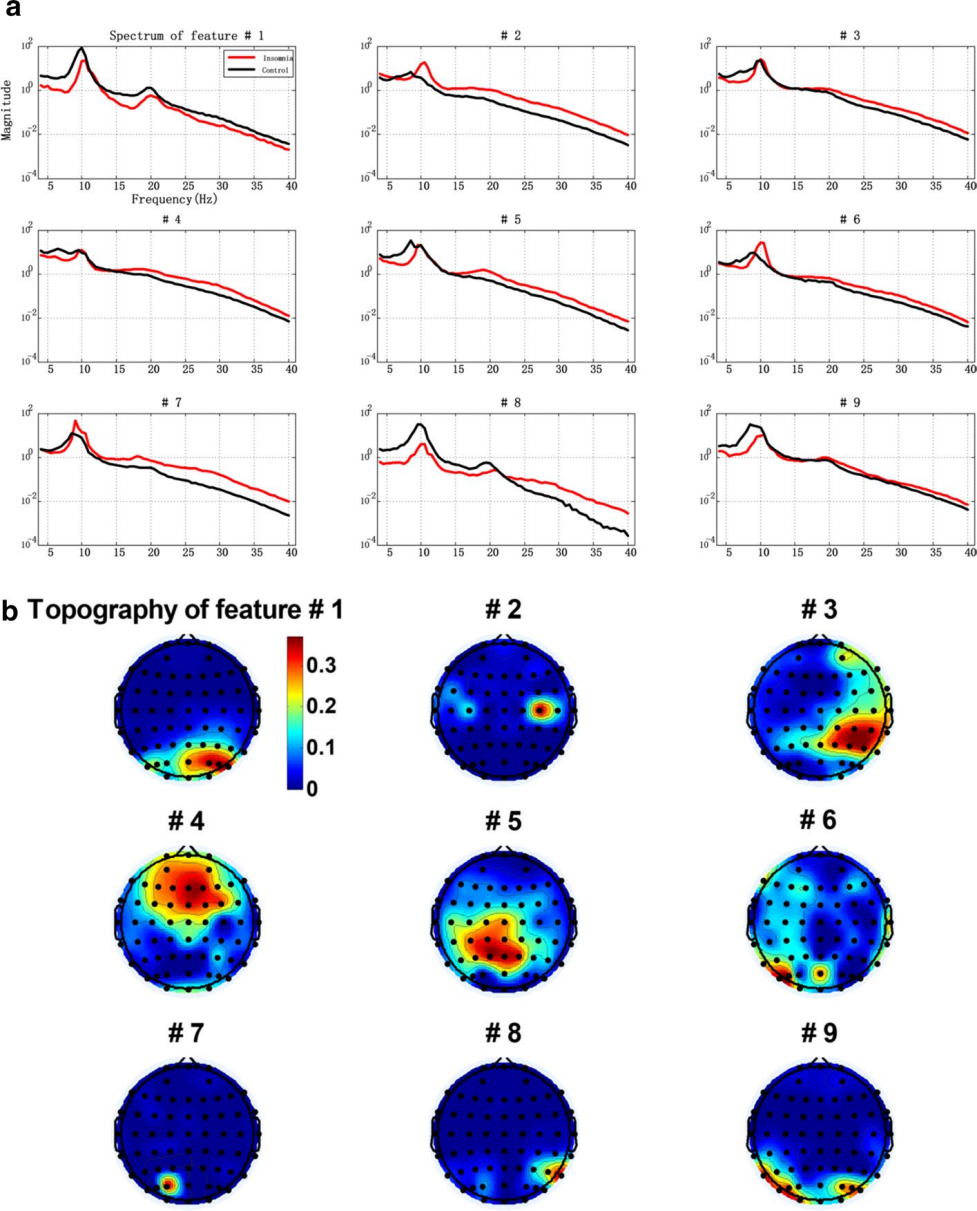
**a** Average of selected spectrum-domain extracted features; **b** their corresponding spatial components extracted by lraNMF_HALS

The spectrum-domain features and corresponding spatial distribution are illustrated in Fig. 7. In Fig. 7a, the power spectrum values were log-transformed. Group difference was examined between insomnia and normal sleepers in absolute spectrum of 4–40 Hz for the component of interest. Insomnia group is noted by red line and normal sleeper group is noted by black line.

**Fig. 8:**
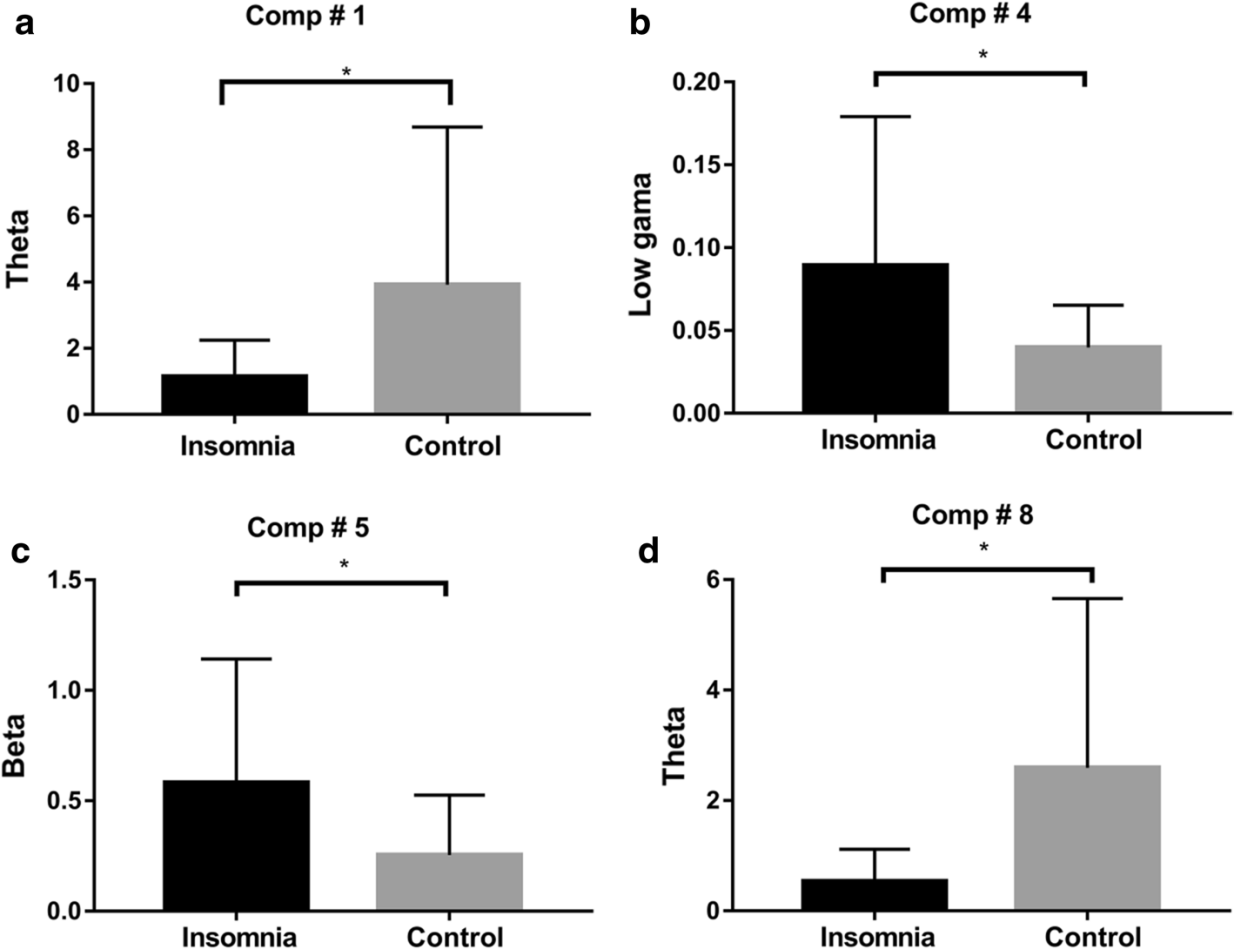
Four components that show significant main effects between insomnia and control. *P < 0.05

Furthermore, the nine features were statistic by two-way *t* test. We found that the feature#1 and #8 revealed a significant main effect in theta band (*P* < 0.05). From Fig. 7b, the #1 and #8 of brain map, the result indicated that the right occipital region has a main effect of group in theta band. The feature #4 also revealed that a significant main effect in low gamma band (30–40 Hz), *P* < 0.05. On the basis of the #4 of the brain maps in Fig. 7b, there was significant difference in low gamma band at frontal lobe. The feature #5 also revealed that a significant main effect in beta band, *P* < 0.05. In accordance with the #5 in the brain map in Fig. 7a, there was the significant difference in beta band at parietal lobe. The significant differences are shown in Fig. 8. For the other five features, no main effect of group was observed.

## Discussion

In this study, we provide a comparison of four NMF algorithms in terms of accuracy of estimation, stability (repeatability of the results) and time complexity of algorithms with simulated data. In the practical application of NMF algorithms, stability plays an important role, which was an emphasis in the comparison. A hierarchical clustering algorithm was implemented to evaluate the stability of NMF algorithms. In simulation, based on comprehensive analysis of fit, stability, accuracy of estimation and time complexity, hierarchical alternating least squares (HALS) low-rank NMF algorithm (lraNMF_HALS) outperformed the other three NMF algorithms. In the application of lraNMF_HALS for real resting-state EEG data analysis, stable and interpretable features were extracted.

Based on our assessment, lraNMF_HALS is the most stable algorithm. In order to guarantee the reliability of estimated components, the model order is selected in terms of algorithm stability. With these strategies stable components were reconstructed. These results of lraNMF_HALS for scalp resting-state EEG data analysis are consistent with the previous findings and also provide more reasonable results in support of pathological mechanisms of insomnia. Corsi-Cabrera et al. found that patients with insomnia exhibited significantly higher gamma power at frontoparietal [28]. This is consistent with the result of Comp#4. Szelenberger et al. pointed out that theta band in insomniacs is lower than normal subjects, but beta band is higher than normal subjects [26]. This is in accordance with the result of the Comp#1 and Comp#5. The consistent results show that the proposed method is effective for spectrum analysis of EEG. In addition, we also found some results that were not found before, which provide support of pathological mechanisms of insomnia. The Comp#4 and Comp#5 with higher power can verify that the wakefulness of the insomniacs is higher than that of the normal subjects, which may lead to their insomnia. Based on the evaluation of algorithm stability, the repeatability of the results is guaranteed. This finding provides theoretical support for clinical treatment of insomnia.

When adaptive algorithm is applied in real-world application, more attention needs to be paid to its stability. Since initialization of adaptive algorithm may be different in different runs, it may converge to local optimum, which makes results different for different runs. It is vital for scientific research. The stability of algorithms could be evaluated with clustering analysis of components extracted from multiple runs of the same algorithm. The study of algorithm stability can not only quantitatively describe the reproducibility of the components, but also provide an effective criterion for the comparison of different algorithms and the selection of model order.

## Conclusion

In this study, we proposed a method to compare different NMF algorithms so as to extract stable components. Specifically, we provide a comparison of four NMF algorithms in terms of accuracy of estimation, stability of algorithms and time complexity with simulated data. The performance of NMF algorithms also evaluated scalp resting-state EEG data analysis from aspects of stability and time complexity. In the application of lraNMF_HALS for real resting-state EEG data analysis, stable and interpretable features were extracted. From both simulation and real-world application, lraNMF_HALS outperformed the other three NMF algorithms in terms of algorithm stability and computational time. In simulation, the comparison is illustrated in Fig. 4. From simulation, we also find that lraNMF_HALS can estimate the most accurate potential components. In real-world application, in terms of computational time, lrNMF_HALS (72.315s) is faster than NMF_MU (1157.007s), HALS (1173.178s) and lraNMF_MU (106.010s) and in terms of algorithms stability, lrNMF_HALS (0.833) is more stable than NMF_MU (0.760), HALS (0.717) and lraNMF_MU (0.622). Based on the results of assessment, our recommendation is to use lraNMF_HALS, providing the most accurate and robust estimation as well as offering an intuitive interpretation. In terms of reproducibility of the final estimated components, this work provides a useful pipeline that applied NMF to spectrum analysis of EEG data. The pipeline includes selection of algorithm, selection of model order and stability analysis of estimated components. Based on the novel pipeline, components related to the pathology of insomnia were extracted and provide new ideas for clinical diagnosis and treatment of insomnia. For further study, the method is worthy of being applied on some other EEG spectrum data and identify more reasonable features. When an investigator prepares to use NMF for subsequent analysis, NMF performance needs to be considered in the experimental design to obtain stable and reliable results. Through this research, we found that different algorithms have a great impact on the decomposition results. In the following research, it is necessary to propose an NMF algorithm specifically applied to EEG spectrum analysis.

## Methods

### NMF algorithms

For a given nonnegative data matrix $V \in {\mathbb{R}}^{m \times n}$ and the number of extracted components $r < \hbox{min} \left[ {m,n} \right]$, NMF attempts to find nonnegative matrices $W \in {\mathbb{R}}^{m \times r}$ and $H \in {\mathbb{R}}^{r \times n}$ which minimize the cost function as follows:

1 $$fW,H = \frac{1}{2}V - WH_{F}^{2} ,$$

where $H$ and $W$ are coefficient matrix and component matrix, respectively. Their product is a rank-r approximate estimation of $V$. In practical applications, the selection of $r$ is critical and has great influence on the results. In this study, the model order, *r*, would be determined by checking the stability of the model with different values:

2 $$V_{m \times n} \approx W_{m \times r} H_{r \times n} , W \ge 0, H \ge 0 .$$

To optimize Eq. (1), Lee and Seung suggested a very popular multiplicative update rule with computational complexity of ${\mathcal{O}}\left( {mnr} \right)$ [6]. The iterative formulas are as follows:

3 $$H \leftarrow H.*\frac{{V^{T} W}}{{HW^{T} W}},$$

4 $$W \leftarrow W.*\frac{VH}{{WH^{T} H}}.$$

In 1998, Rasmus Bro proposed another set of updated formula [56] which is a column-wise method. And then it was extended with HALS algorithm and it can be computed in the complexity of ${\mathcal{O}}\left( {mn} \right)$ [2], where the columns of $H$ and $W$ are updated sequentially:

5 $$h_{i} \leftarrow \frac{1}{{w_{i}^{T} w_{i} }}\left[ {V_{i}^{T} w_{i} } \right],$$

6 $$w_{i} \leftarrow \frac{1}{{h_{i}^{T} h_{i} }}\left[ {V_{i} h_{i} } \right] ,$$

where $V_{i} = V - \mathop \sum \nolimits_{j \ne i} w_{j} h_{j}^{T}$. $w_{j}$ and $h_{j}$ are the ${\text{jth}}$ column of $W$ and $H$, respectively. Only one column of $W$ and $H$ are updated in each iteration of HALS algorithm. Since $r$ columns included in $W$ and $H$, essentially, NMF_MU and HALS have equivalent space complexity and time complexity. However, in practical application, HALS is usually faster than NMF_MU [57].

Although HALS is faster than NMF_MU, the two algorithms still have a lot of room for improvement in computing speed. The common bottleneck of the computing speed is the size of matrix $V$. In the process of updating $W$ and $H$, the large original data $V$ will be used for many times. This issue causes not only slow convergence, but also great consuming of computer memory. To address the issue is to replace the large matrix with a smaller one. Then the efficiency of HALS and NMF_MU could be improved. Motivated by the intuition, low-rank approximation (LRA) based NMF was proposed with computational complexity of ${\mathcal{O}}\left( {nr^{2} } \right)$ [57]:

7 $$\begin{array}{*{20}c} {\hbox{min} } \\ {\overset{\lower0.5em\hbox{$\smash{\scriptscriptstyle\frown}$}}{W} ,\;\overset{\lower0.5em\hbox{$\smash{\scriptscriptstyle\frown}$}}{H} ,\;W ,\;H} \\ \end{array} \;F\;\left( {\overset{\lower0.5em\hbox{$\smash{\scriptscriptstyle\frown}$}}{W} ,\;\overset{\lower0.5em\hbox{$\smash{\scriptscriptstyle\frown}$}}{H} ,\;W,\;H} \right)\;{ = }\;\begin{array}{*{20}c} {\hbox{min} } \\ {\overset{\lower0.5em\hbox{$\smash{\scriptscriptstyle\frown}$}}{W} ,\;\overset{\lower0.5em\hbox{$\smash{\scriptscriptstyle\frown}$}}{H} ,\;W,\;H} \\ \end{array} \left( {\parallel V\; - \;\overset{\lower0.5em\hbox{$\smash{\scriptscriptstyle\frown}$}}{W} \;\overset{\lower0.5em\hbox{$\smash{\scriptscriptstyle\frown}$}}{H}^{T} \parallel_{F}^{2} \; + \;\parallel \overset{\lower0.5em\hbox{$\smash{\scriptscriptstyle\frown}$}}{W} \;\overset{\lower0.5em\hbox{$\smash{\scriptscriptstyle\frown}$}}{H}^{T} \; - \;WH^{T} \;\parallel_{F}^{2} } \right),$$

where $$W \in {\mathbb{R}}_{ + }^{m \times r}$$, $$H \in {\mathbb{R}}_{ + }^{r \times n}$$, $$\overset{\lower0.5em\hbox{$\smash{\scriptscriptstyle\frown}$}}{W} \; \in \;{\mathbb{R}}_{ + }^{{m \times {\text{l}}}}$$, $$\overset{\lower0.5em\hbox{$\smash{\scriptscriptstyle\frown}$}}{H} \; \in \;{\mathbb{R}}_{ + }^{{N\; \times \;{\text{l}}}}$$, $$l = {\text{pr}} \ll m$$, and $$p$$ is a small positive constant. In order to solve Eq. (7), the cost function $$\begin{array}{*{20}c} {\hbox{min} } \\ {\overset{\lower0.5em\hbox{$\smash{\scriptscriptstyle\frown}$}}{W} ,\;\overset{\lower0.5em\hbox{$\smash{\scriptscriptstyle\frown}$}}{H} ,\;W,\;H} \\ \end{array} \;\parallel V\; - \;\overset{\lower0.5em\hbox{$\smash{\scriptscriptstyle\frown}$}}{W} \;\overset{\lower0.5em\hbox{$\smash{\scriptscriptstyle\frown}$}}{H}^{T} \parallel_{F}^{2}$$ was used, where $$\overset{\lower0.5em\hbox{$\smash{\scriptscriptstyle\frown}$}}{W}$$ and $$\overset{\lower0.5em\hbox{$\smash{\scriptscriptstyle\frown}$}}{H}^{T}$$ are with the low-rank $${\text{l}}$$, $$l \ll m$$. Then, optimize $$\parallel \overset{\lower0.5em\hbox{$\smash{\scriptscriptstyle\frown}$}}{W} \;\overset{\lower0.5em\hbox{$\smash{\scriptscriptstyle\frown}$}}{H}^{T} \; - \;W\;H^{T} \parallel_{F}^{2}$$ with fixed $$\overset{\lower0.5em\hbox{$\smash{\scriptscriptstyle\frown}$}}{W}$$ and $$\overset{\lower0.5em\hbox{$\smash{\scriptscriptstyle\frown}$}}{H}^{T}$$.

The prototypical low-rank NMF algorithms originated by Guoxu Zhou and Andrzej Cichocki [57] are provided as follows:

8 $$H \leftarrow H.*\frac{{\left[ {\overset{\lower0.5em\hbox{$\smash{\scriptscriptstyle\frown}$}}{H} \left( {\overset{\lower0.5em\hbox{$\smash{\scriptscriptstyle\frown}$}}{W}^{T} W} \right)} \right]}}{{H\left( {W^{T} W} \right)}},$$

9 $$W \leftarrow W.*\frac{{\left[ {\overset{\lower0.5em\hbox{$\smash{\scriptscriptstyle\frown}$}}{W} \;\left( {\overset{\lower0.5em\hbox{$\smash{\scriptscriptstyle\frown}$}}{H}^{T} H} \right)} \right]}}{{W\left( {H^{T} H} \right)}} .$$

This is lraNMF_MU that low-rank approximation-based multiplicative update (NMF_MU). The initialization of $$\overset{\lower0.5em\hbox{$\smash{\scriptscriptstyle\frown}$}}{W}$$ and $$\overset{\lower0.5em\hbox{$\smash{\scriptscriptstyle\frown}$}}{H}$$ come from PCA singular value decomposition (tSVD). In the first step, suppose that the optimal $$\overset{\lower0.5em\hbox{$\smash{\scriptscriptstyle\frown}$}}{W}$$ and $$\overset{\lower0.5em\hbox{$\smash{\scriptscriptstyle\frown}$}}{H}^{T}$$, i.e., $${\text{V}}\; \approx \;\overset{\lower0.5em\hbox{$\smash{\scriptscriptstyle\frown}$}}{W} \;\overset{\lower0.5em\hbox{$\smash{\scriptscriptstyle\frown}$}}{H}^{T}$$. Then above function would be used to optimize the cost function $$\hbox{min} \parallel \overset{\lower0.5em\hbox{$\smash{\scriptscriptstyle\frown}$}}{W} \;\overset{\lower0.5em\hbox{$\smash{\scriptscriptstyle\frown}$}}{H}^{T} - {\text{WH}}^{\text{T}} \parallel_{F}^{2}$$. At first sight, there is no great difference between Eqs. (3) and (8). But note that the dimensions of $$\overset{\lower0.5em\hbox{$\smash{\scriptscriptstyle\frown}$}}{W}$$ and $$\overset{\lower0.5em\hbox{$\smash{\scriptscriptstyle\frown}$}}{H}^{T}$$ are much smaller than that of $$V$$. Under the present circumstances,$${\text{l}} = {\text{pr}} \ll m$$, lraNMF_MU has much lower space and time complexity.

Similar to the HALS algorithm, let $$V_{i} = \overset{\lower0.5em\hbox{$\smash{\scriptscriptstyle\frown}$}}{W} \;\overset{\lower0.5em\hbox{$\smash{\scriptscriptstyle\frown}$}}{H}^{T} \; - \,\mathop \sum \nolimits_{j \ne i} w_{j} h_{j}^{T}$$ and Eqs. (5, 6) become:

10 $$h_{i} \leftarrow \frac{1}{{w_{i}^{T} w_{i} }}\left[ {\overset{\lower0.5em\hbox{$\smash{\scriptscriptstyle\frown}$}}{H} \left( {\overset{\lower0.5em\hbox{$\smash{\scriptscriptstyle\frown}$}}{W}^{T} w_{i} } \right)\; - \;\overline{{H_{1} }} \left( {\overline{{W_{1} }}^{T} w_{i} } \right)} \right],$$

11 $$w_{i} \leftarrow \frac{1}{{h_{i}^{T} h_{i} }}\left[ {\overset{\lower0.5em\hbox{$\smash{\scriptscriptstyle\frown}$}}{W} \left( {\overset{\lower0.5em\hbox{$\smash{\scriptscriptstyle\frown}$}}{H}^{T} h_{i} } \right)\; - \;\overline{{W_{1} }} \left( {\overline{{H_{1} }}^{T} h_{i} } \right)} \right] ,$$

where $\overline{{W_{1} }} \in {\mathbb{R}}^{{m \times \left( {r - 1} \right)}}$ and $\overline{{H_{1} }} \in {\mathbb{R}}^{{{\text{n}} \times {\text{r}} - 1}}$ are the submatrices of $W$ and $H$ by removing their ${\text{ith}}$ column. The low-rank approximation-based HALS is named as lraNMF_HALS, which could be computed in the complexity of ${\mathcal{O}}\left( {nr^{ } } \right)$ [57].

We also provide a summary of the comparison of different NMF algorithms that have been studied by the various published methods, as listed in Table 1, which may serve as a reference of method selection according to the available data types of the analyzers.

**Table 1:** Comparison of four NMF algorithms

Algorithms	Cost function	Iteration	Advantages
NMF_MU	$fW,H = \frac{1}{2}\parallel V - WH\parallel_{F}^{2}$	$H \leftarrow H.*\frac{{V^{T} W}}{{HW^{T} W}}$ $W \leftarrow W.*\frac{VH}{{WH^{T} H}}$	The original realization of NMF
HALS		$h_{i} \leftarrow \frac{1}{{w_{i}^{T} w_{i} }}\left[ {V_{i}^{T} w_{i} } \right]$ $w_{i} \leftarrow \frac{1}{{h_{i}^{T} h_{i} }}\left[ {V_{i}^{ } h_{i} } \right]$	Iteration column by column, faster than NMF_MU in practical application
lraNMF_MU	$$F\;\left( {\overset{\lower0.5em\hbox{$\smash{\scriptscriptstyle\frown}$}}{W} ,\;\overset{\lower0.5em\hbox{$\smash{\scriptscriptstyle\frown}$}}{H} ,\;W,\;H} \right)\; = \;\parallel V\; - \;\overset{\lower0.5em\hbox{$\smash{\scriptscriptstyle\frown}$}}{W} \overset{\lower0.5em\hbox{$\smash{\scriptscriptstyle\frown}$}}{H}^{T} \parallel_{F}^{2} \; + \;\parallel \overset{\lower0.5em\hbox{$\smash{\scriptscriptstyle\frown}$}}{W} \overset{\lower0.5em\hbox{$\smash{\scriptscriptstyle\frown}$}}{H}^{T} \; - \;WH^{T} \parallel_{F}^{2}$$	$$H \leftarrow H.*\frac{{\left[ {\overset{\lower0.5em\hbox{$\smash{\scriptscriptstyle\frown}$}}{H} \left( {\overset{\lower0.5em\hbox{$\smash{\scriptscriptstyle\frown}$}}{W}^{T} W} \right)} \right]}}{{H\left( {W^{T} W} \right)}}$$ $$W \leftarrow W.*\frac{{\left[ {\overset{\lower0.5em\hbox{$\smash{\scriptscriptstyle\frown}$}}{W} \left( {\overset{\lower0.5em\hbox{$\smash{\scriptscriptstyle\frown}$}}{H}^{T} H} \right)} \right]}}{{W\left( {H^{T} H} \right)}}$$	Dimension reduction at the start of the algorithm. Fix the problem of the size of matrix to be decomposed. Faster than NMF_MU in theory and in practical application
lraNMF_HALS		$$h_{i} \leftarrow \frac{1}{{w_{i}^{T} w_{i} }}\left[ {\overset{\lower0.5em\hbox{$\smash{\scriptscriptstyle\frown}$}}{H} \left( {\overset{\lower0.5em\hbox{$\smash{\scriptscriptstyle\frown}$}}{W}^{T} w_{i} } \right)\; - \;\overline{{H_{i} }} \left( {\overline{{W_{i} }}^{T} w_{i} } \right)} \right]$$ $$w_{i} \leftarrow \frac{1}{{h_{i}^{T} h_{i} }}\left[ {\overset{\lower0.5em\hbox{$\smash{\scriptscriptstyle\frown}$}}{W} \left( {\overset{\lower0.5em\hbox{$\smash{\scriptscriptstyle\frown}$}}{H}^{T} h_{i} } \right) - \;\overline{{W_{i} }} \left( {\overline{{H_{i} }}^{T} h_{i} } \right)} \right]$$	Dimension reduction at the start of the algorithm. Faster than HALS in theory and practical application

### Fitness of the algorithm

The *fit* is defined as follows:

12 $${\text{fit}}\left( {V,\;\overset{\lower0.5em\hbox{$\smash{\scriptscriptstyle\frown}$}}{V} } \right)\; = \;1\; - \;\frac{{\parallel V - \overset{\lower0.5em\hbox{$\smash{\scriptscriptstyle\frown}$}}{V}_{F} \parallel }}{{\parallel V_{F} \parallel }} ,$$

where $$\overset{\lower0.5em\hbox{$\smash{\scriptscriptstyle\frown}$}}{V}$$ is a reconstructed version of V by Eq. (2). Apparently, fit (V, $$\overset{\lower0.5em\hbox{$\smash{\scriptscriptstyle\frown}$}}{V}$$) = 1 if and only if $$\overset{\lower0.5em\hbox{$\smash{\scriptscriptstyle\frown}$}}{V} = V$$ [57]. In the previous study, *fit* was used as the performance index of decomposition [2]. However, we find that even when the *fits* of two NMF decomposition are similar, the extracted components could be different. Hence the fit is not enough to evaluate the stability of the decomposition.

### Hierarchical clustering

Hierarchical clustering is one of most popular clustering methods. In contrast to partitioned clustering, which directly decomposes the features into a set of disjoint clusters, the hierarchical clustering method is the process for transforming a proximity matrix into a nested partition, which can be graphically represented by a tree called dendrogram. Cutting the tree at different selected heights will provide a partitioning cluster at selected precision. So the precision is the one that is “tuned” by the cut [58]. This algorithm was applied to validate the stability of ICA components and it was named ICASSO [59].

Hierarchical clustering algorithms have two different types: agglomerative clustering (bottom-up) and divisive clustering (top-down). In this study, agglomerative clustering was used, and the dendrogram is formed from bottom to up. For this clustering method, at the first iteration, the number of clusters is same as the number of objects N. At the second iteration, the most similarity cluster will merge as a new cluster, so the number of clusters will become *N*-1. At the third iteration, the number of clusters will become *N*-2. As the clustering goes forward, the number of clusters will become 1. By this way, the relation dendrogram of different objects could be established. In the stability algorithm, the cluster result is the dendrogram at the level that is the number of extracted features of NMF.

A conservative cluster quality index $I_{q}$ in the previous study [59] was defined to reveal the compactness and isolation of a cluster. It is computed as the difference between the average intra-cluster similarities and average extra-cluster similarities:

13 $${\text{I}}_{\text{q}} \left( {{\text{C}}_{\text{m}} } \right)\; = \;\frac{1}{{\left| {C_{m} } \right|^{2} }}\mathop \sum \limits_{{i,j \in C_{m} }} \sigma_{ij} - \frac{1}{{\left| {C_{m} } \right|\left| {C_{ - m} } \right|}}\mathop \sum \limits_{{i \in C_{m} }} \mathop \sum \limits_{{j \in C_{ - m} }} \sigma_{ij} ,$$

where $C_{m}$ means the features of *m*th cluster. $\sigma_{ij}$ is the similarity of the *i*th and *j*th features. $C_{ - m} \; = \;C\; - \;C_{m}$ represent the features that not belong to this cluster. Ideally, the same component from different runs will be clustered in the same cluster. The different components are in different clusters. The number of clusters equals to the number of extracted components. The number of features in each cluster is the number of runs of the algorithm. First term $\frac{1}{{\left| {C_{m} } \right|^{2} }}\mathop \sum \nolimits_{{i,j \in C_{m} }} \sigma_{ij}$ is the average similarity of *m*th cluster (average intra-cluster similarities). This part is used to describe the similarity of the components in the same cluster. Second term $\frac{1}{{\left| {C_{m} \parallel \left. {C_{{{-}m}} } \right|} \right.}}\;\mathop \sum \nolimits_{{i\; \in \;C_{m} }} \mathop \sum \limits_{{j\; \in \;C_{{{-}m}} }} \sigma_{ij}$ is the average similarity between the features that belong to *m*th cluster and not belong to *m*th cluster (average extra-cluster similarities). This part is used to describe the dissimilarity of the components in different clusters. The range of $I_{q}$ is from 0 to 1. The value closer to 1, the higher stability it is.

### Evaluation of stability of NMF algorithms

Given a non-negative data matrix $V \in {\mathbb{R}}_{ + }^{m \times n}$, we used an NMF algorithm to decompose the data. In order to evaluate the stability and reliability of components decomposed by NMF, three steps are used, which is introduced in our previous study [60]. Three steps are as follows:

Step 1: An NMF algorithm was run K times. Each time with random initialization.

Step 2: All extracted components were clustered. According to their mutual similarities, agglomerative clustering was used.

Step 3: The centroid of each cluster was selected as the component extracted by NMF. The cluster quality index $I_{q}$ works as stability index for each component.

## Data Availability

The data sets used and/or analyzed during the current study are available from the corresponding author on reasonable request.

## Abbreviations

NMF: Nonnegative matrix factorization
EEG: Electroencephalography
PMF: Positive matrix factorization
BOLD: Blood oxygen level dependent
ICA: Independent component analysis
PCA: Principal component analysis
BCI: Brain computer interface
BSS: Blind sources separation
NMF_MU: NMF-based multiplicative update
HALS: Hierarchical alternating least squares
lraNMF_MU: Low-rank approximation-based multiplicative update
lraNMF_HALS: Hierarchical alternating least squares (HALS) low-rank NMF algorithm
